# Is there a link between IL-23/IL-17 and developmental pathways such as the Wnt and Hedgehog pathway?

**DOI:** 10.31138/mjr.28.1.59

**Published:** 2017-03-28

**Authors:** Nicholas Stamatis-Liossis, Dimitrios Daoussis, ASSOCIATED CENTRES/CLINICS, Alexandros Drosos, Lazaros Sakkas

**Affiliations:** University Department of Internal Medicine, Rheumatology Department, Rheumatology Research Laboratory, Medical University of Patras, Greece; Department of Rheumatology, Medical University of Ioannina; Rheumatology Department, University Medical School of Larissa

**Keywords:** IL-23, IL-17, Wnt, Hedgehog, pathways, ankylosing spondylitis

## Abstract

Recent experimental evidence suggests that IL-23 may induce spondyloarthropathy by acting on entheseal resident “innate-like” T cells. These cells express IL-23R and respond to IL-23 by secreting inflammatory cytokines such as IL-6 and IL-17 as well as IL-22 which acts on osteoblasts and regulates bone remodeling. Moreover, a large amount of evidence indicates that new bone formation in the form of osteophytes is mainly driven by reactivation of developmental pathways such as the Wnt and the Hedgehog pathway. We hypothesize that IL-23/IL-17 may mediate bone remodeling by affecting the expression of developmental pathways.

## METHODOLOGY

We will perform a series of experiments to assess the potential effect of IL-22, IL-23, IL-17 and IL-6 on the Wnt and Hedgehog pathways in human osteoblasts, osteoblast-like cell lines (such as Saos2) and human chondrocytes. We will first explore whether the above-mentioned cells and cell lines express the relevant receptors by using immunocytochemistry. Then, we will treat these cells/cell lines with IL-22, IL-23, IL-17 and IL-6 and then assess the activation of the Wnt and Hedgehog pathway. Time and dose response experiments will be performed for each cytokine. Western blot analysis for active β-catenin and RT-PCR for Ptch-1, Gli1 and Gli2 will be used to assess the activation of the Wnt and Hedgehog pathway, respectively.

## INTRODUCTION-BACKGROUND

Ankylosing spondylitis (AS) is a part of a heterogeneous group of diseases called spondyloarthropathies. One of the hallmarks of AS is new bone formation, eventually leading to joint ankylosis and functional impairment. Non-steroidal anti-inflammatory drugs (NSAIDs) are the main therapeutic option for axial involvement in AS; these drugs are quite effective, but still, a significant proportion of patients do not respond to these agents. TNF blockers are highly effective in controlling inflammation in patients with AS; however, it is not known whether these agents are able to retard radiographic progression and inhibit new bone formation. Clinical trials have shown that TNF blockers do not inhibit the process of new bone formation, at least when administered up to 2 years.^1–2^

During the last years, accumulating evidence points to the direction that new bone formation in AS is mainly controlled by developmental pathways such as the Wnt or the Hedgehog (HH) pathway. Developmental pathways are mainly active during embryogenesis; however, they remain active during adult life and participate in homeostatic functions such as repair following injury.^3–5^

A great amount of experimental evidence indicates that the canonical Wnt pathway (also known as β-catenin pathway) is a critical regulator of osteoblastogenesis.^6–8^ Our research group has extensively studied the role of the Wnt pathway in the process of new bone formation in AS. We have previously shown that Dickkopf-1, an inhibitor of the Wnt pathway, is dysfunctional in AS: a finding with potential pathogenetic implications.^9^

The HH pathway is considered to be the main controller of endochondral ossification;^10^ it is noteworthy that osteophytes are mainly produced through this process. Experimental data indicate that HH pathway inhibition in animal models of osteoarthritis (OA) reduced osteophyte formation;^11^ the same was true for animal model of arthritis.^12^ These data point to the direction of a link between HH pathway activation and osteophyte formation.

Recently, there has been a major breakthrough in the pathophysiology of spondyloarthropathies: a vast amount of clinical and experimental evidences supports a fundamental role of the IL-17/IL-23 axis in pathogenesis. A landmark study published in Nature Medicine a few years ago found that entheses actually have immune cells that express the receptor of IL-23.^13^ These cells are characterized as innate like lymphoid cells (ILC) and express RORgt and CD3, but not CD4 or CD8. In animal models, IL-23 overexpression using minicircle technology leads to a phenotype that resembles spondyloarthropathies. This animal model led to a new concept for SpA pathogenesis. IL-23 overexpression (presumably from the gut) leads to activation of ILCs in entheses which produce a great amount of cytokines such as IL-17 and IL-22; these cytokines seem to drive many manifestations of the disease (IL-17 is linked to systemic inflammation and IL-22 to new bone formation).^14^

In humans, the main proof that the IL-17/IL-23 axis plays a central pathogenetic role comes from clinical studies showing significant clinical efficacy of IL-17 and IL-23 blockers in spondyloarthropathies.

## PURPOSE OF THE STUDY

This study aims to:

1. Assess whether IL-23 is over expressed in patients with AS; and
2. Assess whether there is a link between IL-23 (or the associated IL-17 and IL-22 cytokines) and developmental pathways such as the Wnt and the HH pathway.

## STUDY DESIGN

This study will have a clinical and a basic research part.

### · Clinical

We are planning to measure IL-23 serum levels in 150–200 patients with AS compared to 100 healthy subjects, age and gender matched.

In all patients, we will record demographics and clinical data such as disease activity score (BASDAI), radiological score (mSASSS) and medications.

This part of the study will address whether IL-23 levels are increased in patients with AS.

### · Basic research

We aim at assessing whether IL-23, IL-22 or IL-17 are able to activate the Wnt or the HH pathway in osteoblasts.

We will first use osteoblast-like Saos2 cells as a model, since these cells are easy to culture and already available at our laboratory. At a later time, we will perform the same experiments on a human osteoblastic cell line (NHost, Clonetics) and human osteoblasts extracted from patients with either OA or AS undergoing joint replacement surgery.

We will start by assessing whether Saos2 and human osteoblasts actually have receptors for IL-23, IL-22 and IL-17 by employing immunocytochemistry using the proper monoclonal antibodies.

The next step will be the assessment of Wnt and HH pathway activation in Saos2 cells following activation with IL-23, IL-22, IL-17, IL-6 and IL-1.

Unstimulated cells cultured under the same conditions will be used as controls.

We will perform time and dose response experiments for each cytokine.

Cells will be cultured on Petri dishes (10^6^ per dish) until they reach 80% confluence. Then, we will add each cytokine at a concentration and time according to relevant dose and time response experiments. Cells will be detached using a chemical method; half of them will be used for protein extraction (BioRad) and the rest for RNA extraction (Quiagen, RNeasy kit).

HH pathway activation will be assessed using RT-PCR. Specifically, we will assess the expression of 3 HH pathway target genes (PTCH1, GLI1 and GLI2).

Wnt pathway activation will be assessed by Western Blot. Specifically, we will assess the expression of active (unphosphorylated) β-catenin (clone 8E7, Millipore) by Western immunoblots.

## DATA ANALYSIS

With these experiments, we will assess whether IL-23/IL-17 are able to activate developmental pathways in osteoblastic cells by comparing results from the control group (unstimulated cells) or cells stimulated by cytokines not linked to the IL-23/IL-17 axis such as IL-6 or IL-1.

## SIGNIFICANCE OF THE STUDY

The main therapeutic targets in AS are to control inflammation, improve quality of life and inhibit radiographic progression/ankylosis. TNF blockers are effective in reducing pain and stiffness and improving overall quality of life. However, they do not seem to retard osteophyte formation. This study aims to explore whether IL-23 is indeed overexpressed in patients with AS, and if this cytokine is able to activate the key molecular pathways involved in new bone formation. If our data support an active role of IL-23/IL-17 in activating these molecular pathways, then clinical research will need to focus on the effect of newer biologics such as secukinumab or ustekinumab on radiographic progression in patients with AS.
